# Molecular Identification, Antifungal Susceptibility, and Geographic Origin of Clinical Strains of *Sporothrix schenckii* Complex in Mexico

**DOI:** 10.3390/jof4030086

**Published:** 2018-07-20

**Authors:** Olga C. Rojas, Alexandro Bonifaz, Christian Campos, Rogelio de J. Treviño-Rangel, Rafael González-Álvarez, Gloria M. González

**Affiliations:** 1 Departamento de Ciencias Básicas, Vicerrectoría de Ciencias de la Salud, Universidad de Monterrey, San Pedro Garza García, Monterrey 66238, Mexico; olga.rojas@udem.edu; 2 Departamento de Microbiología, Facultad de Medicina, Universidad Autónoma de Nuevo León, Monterrey 64460, Mexico; cliscamposc@gmail.com (C.C.); roghe24@gmail.com (R.d.J.T.-R.); 3 Departamento de Micología & Servicio de Dermatología, Hospital General de México, México City 06726, Mexico; a_bonifaz@yahoo.com.mx; 4 Departamento de Genética y Medicina Genómica, Facultad de Medicina, Universidad Autónoma de Guadalajara, Zapopan 45129, Mexico; rgonzaleza5@hotmail.com

**Keywords:** *Sporothrix schenckii*, *Sporothrix globosa*, CAL gen, clinical, origin, Mexico

## Abstract

Sporotrichosis is a subcutaneous mycosis caused by *Sporothrix schenckii* complex. The disease has been reported worldwide. However, the incidence of the etiological agent varies in its geographic distribution. We studied 39 clinical isolates of *Sporothrix schenckii* from diverse regions in Mexico, collected from 1998 to 2016. Molecular identification was performed by sequence analysis of the partial calmodulin gene. In vitro antifungal susceptibility to amphotericin B (AMB), itraconazole (ITC), voriconazole (VRC), posaconazole (PSC), fluconazole (FLC), terbinafine (TRB), caspofungin (CSF), anidulafungin (ANF), and micafungin (MCF) was evaluated. Thirty-eight isolates of *S*. *schenckii* complex were divided into five supported clades in a phylogenetic tree. The predominant clinical form was lymphocutaneous (92.3%), fixed cutaneous (5.1%), and disseminated (2.5%). Terbinafine exhibited the best in vitro antifungal activity, while fluconazole was ineffective against *Sporothrix schenckii* complex. Our results showed diverse geographic distribution of clinical isolates in eight states; definitive identification was done by CAL gen PCR-sequencing. In Mexico, *S*. *schenckii* is considered to be an etiological agent of human sporotrichosis cases, and lymphocutaneous is the most prevalent form of the disease. This study revealed four clades of *S*. *schenckii* *sensu stricto* by phylogenetic analysis. Furthermore, we report one case of *S*. *globosa* isolated from human origin from the North of Mexico.

## 1. Introduction

Human sporotrichosis is a subcutaneous mycosis caused by dimorphic fungi *Sporothrix schenckii* species complex, which comprises four species of clinical importance: *S*. *brasiliensis*, *S*. *globose*, *S*. *luriei,* and *S*. *schenckii sensu stricto* [1]. Sporotrichosis has been reported to be endemic in areas of Latin America, South Africa, India, and Japan. In Latin America, the endemic areas are restricted to Peru, Brazil, Mexico, Uruguay, Costa Rica, Guatemala, Colombia, and Venezuela. Sporotrichosis has emerged as a major fungal infection over the last two decades due to changes in epidemiology distribution, taxonomic evolution, and multiple outbreaks. Sporotrichosis in Mexico occurs primarily in regions with a tropical and humid climate. While cases have been reported throughout the country, the highest prevalence has been noted in two zones: the states of Jalisco and Puebla [2]. *Sporothrix* species differ in their geographical distribution. In this sense, *S*. *brasiliensis* is an emerging species restricted to Brazil, and it is highly pathogenic to humans and animals [3]. *S*. *globosa* is distributed worldwide, including Europe, the United States, South America, and Asia [2]. A study of clinical isolates from Northeast China described two clades for *S*. *globosa*—subclade I and subclade II—and found they were not related to geography [4]. In another study from Japan, *S*. *globosa* was divided into two subclades and *S*. *schenkii sensu stricto* strains into three subclades [5]. *Sporothrix luriei* has been reported in three human infections in Africa, Italy, and India [2]. Y. Zhang et al. showed five supported subgroups of *S*. *schenckii* by CAL gen, which was also recognizable in AFLP data [6]. Molecular and phylogenetic analyses of *S*. *schenckii* complex revealed some genetic diversity [7,8]. These species have distinct virulence profiles, and they are rarely identified from clinical origins [5]. The different clinical forms are associated with different species [4]. Sporotrichosis, restricted to skin, is treated by systemic chemotherapy with potassium iodide. Other drugs commonly used are itraconazole for lymphocutaneous infections and amphotericin B for disseminated cutaneous recurrent, extracutaneous, and lymphocutaneous infection [5,8]. The variability in therapeutic efficacy and in vitro activity demonstrated in different studies is attributed to the fact that *S*. *schenckii* is a complex of different species [9,10,11]. Herein, we present Mexican clinical isolates identified by phenotypic characterization and partial sequences of the CAL gen. The demographic characteristics of all stains from diverse regions in Mexico is shown. The antifungal susceptibility patterns of these isolates were also determined in this study.

## 2. Materials and Methods

Thirty-nine clinical isolates were collected between 1998 and 2016 from different Mexican regions (Table 1). All isolates were obtained from a collection of the Microbiology Department of the School of Medicine, Universidad Autónoma de Nuevo León. The isolates were previously identified as *S*. *schenckii* by morphology as well as macroscopic and microscopic features. All isolates had the data regarding the origin of patients and type of lesion.

### 2.1. Phenotypic Characterization

Thirty-nine clinical isolates were re-examined by macroscopic and microscopic studies according to Marimon criteria [12]. Macroscopic characteristics of colonies were studied by culturing isolates on potato dextrose agar (PDA); plates were incubated at 30, 35, and 37 °C. Petri dishes were inoculated with colonies of each fungus and 10 μL of conidial suspension adjusted to 1 × 10^7^ cells/mL for each isolate. After 14 and 21 days, colony diameters were measured in duplicate. Microscopic features of conidia were determined by slide cultures made on PDA after 12–15 days of incubation in the dark and in a humid chamber. Coverslips were mounted in lactophenol cotton blue. The slides were examined under a Nikon Eclipse 50i microscope fitted with a Nikon digital sight DS-2Mv camera. The conidia were measured. The ability of all isolates to reverse to yeast-like cells at 37 °C was performed on brain heart infusion (BHI) with blood for 9 days. Carbohydrate assimilation was tested using API 20C AUX strips (bioMerieux, Mexico City, Mexico) [13].

### 2.2. Antifungal Susceptibility

The antifungal susceptibility test was performed according to the Clinical and Laboratory Standards Institute (CLSI) M38-A2 broth microdilution method [14]. Briefly, a drug stock solution was prepared by dissolving an appropriate amount of amphotericin B (AMB; Bristol Myers Squibb, Princeton, NJ, USA), itraconazole (ITC; Wako Pure Chemicals, Osaka, Japan), voriconazole (VRC, Pfizer, Inc., New York, NY, USA), posaconazole (PSC; Merck, Rahway, NJ, USA), fluconazole (FLC; Pfizer, Inc, Amoise, France), terbinafine (TRB; Novartis, Mexico City, Mexico), caspofungin (CSF; Merk, Rahway, NJ, USA), anidulafungin (ANF; Ben Venve, Northfield Road Bedfor, OH, USA), and micafungin (MCF; Astellas, Tokyo, Japan). The antifungal concentration ranges were adjusted as follows: 0.125 to 16 µg/mL for AMB, ITC, and VRC; from 0.0313 to 16 µg/mL for PSC; from 0.125 to 64 µg/mL for FLC; from 0.004 to 2 µg/mL for TRB; and from 0.015 to 8 µg/mL for CFS, ANF, and MCF. The isolates were cultivated on PDA for 7 days at 30 °C; an inoculum was prepared as recommended by the CLSI and adjusted (80–82% T, λ = 530 nm). The microplates were incubated at 30 °C and read at 72 h. The minimal inhibitory concentration (MIC) endpoint for the triazoles, AMB, MCF, ANF, and TRB was defined as the lowest concentration that produces complete inhibition of growth; for FLC and CSF, it was defined as the lowest drug concentration able to inhibit 50% of visible fungal growth. For quality control of antifungal susceptibilities testing, *Candida albicans* ATCC 90028, *Candida parapsilosis* ATCC 22019, and *Paecilomyces variotii* MYA 3630 were used.

### 2.3. Molecular Identification

Genomic DNA of isolates was extracted from the mycelial phase of the strain on PDA plates at 30 °C for 10 days. DNA was extracted twice with phenol–chloroform and isoamyl–alcohol according to our previous study [15]. Amplification of the partial calmodulin (CAL) gene was performed using the degenerated primers sense CL1 5′-GA(GA)T(AT)CAAGGAGGCCTTCTC-3′ and antisense CL2A 5′-TTTTTGCATCATGAGTTGGAC-3′ [16]. Amplifications were performed in standard condition: one initial cycle of 3 min at 94 °C followed by 30 cycles of 30 s at 94 °C, 50 s at 53.4 °C, 1 min at 72 °C, and a single extension cycle at 72 °C for 5 min. Amplicons were purified using QIAquick (Qiagen, Hilden, Germany) and sequenced in a Genetic Analyzer 3100 (Applied Biosystems, Foster City, CA, USA). Sequences were finally edited in Codon code aligner 4.2.7 software and compared in NCBI GenBank sequences using the BLAST program (http://www.ncbi. nlm.nih.gov/BLAST) to determine their identity. Alignments were performed using Clustal Omega software [17]. A phylogenetic tree was built using the neighbor-joining (NJ) method and a bootstrap analysis [18] of 1000 replicas implemented in MEGA 6.06 software [19]. The phylogenetic tree was constructed with partial calmodulin gene sequences encoding calmodulin-encoding gene [18,19]. The analysis of Sporothrix CAL-related sequences was performed using the sequences obtained in this work (Table 1) and those retrieved from Genbank—*Sporothrix schenckii* (KT427643 and KT427645), *Sporothrix globosa* (KT427636 and KP101459), *Sporothrix brasiliensis* (KJ769111), and *Sporothrix luriei* (KT427639); *Sporothrix mexicana* (KR269843) was used as the out-group.

## 3. Results

### 3.1. Phenotypic Characterization

**Figure 1:**
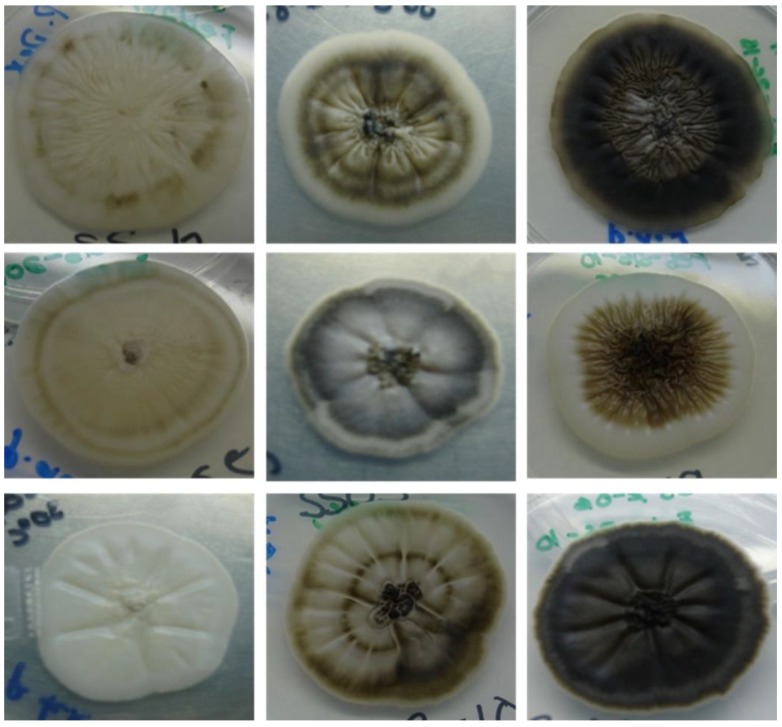
Representative images of colonial morphology of the *Sporothrix schenckii* complex clinical isolates evaluated in this study.

**Figure 2:**
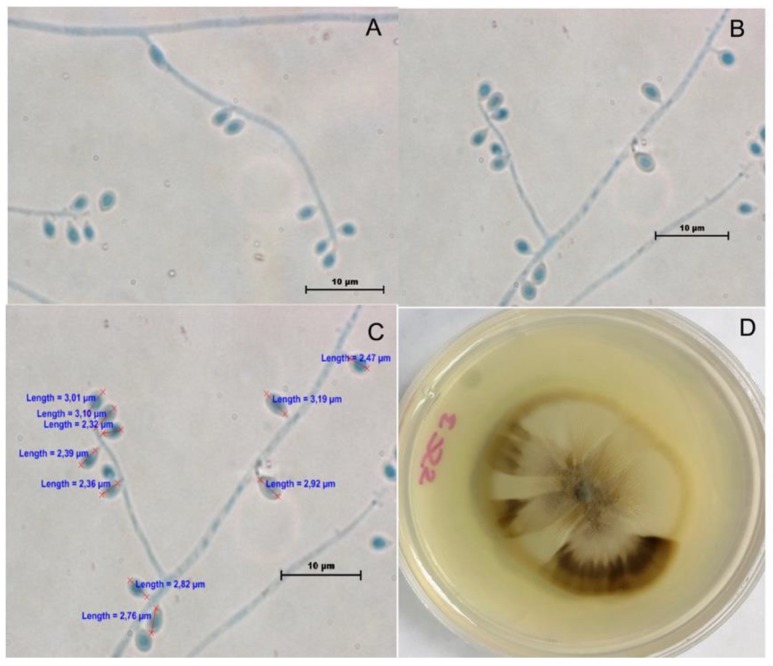
*S*. *globosa* morphological characteristics. (**A**) Hyphae, conidiophore, and sessile conidia (200×); (**B**) Sessile conidia (200×); (**C**) Morphometric analysis (200×); (**D**) Colony on potato dextrose agar (PDA) at 30 °C in 21 days.

A total of 39 clinical isolates from the Microbiology Department collection were studied. The phenotypic characteristic studied as macroscopic morphologies of all isolates were variable after 21 days of incubation. The colonies were initially white/cream colored and later turned brown, then dark brown and with a mixed color on PDA (Figure 1). The colony diameters on PDA after 21 days of incubation attained 30.9 ± 5.0 mm at 30 °C, 11.5 ± 5.3 mm at 35 °C, and 5 ± 1.8 mm at 37 °C. Microscopic features, conidium size, thermotolerance, dimorphism, and the urease test were performed. All isolates showed temperature dimorphism and were urease test positive; conidia were hyaline, ovoid, thick-walled, and almost all isolates were similar. Only three isolates were misidentified as *S*. *globosa* by microscopic features. They exhibited intercalary or terminal conidia formed by sympodial growth from differentiated conidiophores; the conidia were hyaline to slightly pigmented. Sessile conidia were sub *globosa* and dematiaceous (Figure 2). A carbohydrate assimilation test for sucrose and raffinose was positive for all isolates except one (MF948695), which was negative for raffinose.

### 3.2. Antifungal Susceptibility

Antifungal susceptibility testing results are presented in Table 2. Nine drugs were tested. The antifungal susceptibilities (MIC ranges) were as follows: AMB = 0.5–4 µg/mL, ITC = 0.125–2 µg/mL, VRC = 0.5–8 µg/mL, PSC = 0.5–8 µg/mL, FLC > 64 µg/mL, TRB = 0.06–1 µg/mL, CSF = 0.5–8 µg/mL, ANF = 0.25–8 µg/mL, and MCF = 0.5–8 µg/mL. Terbinafine showed the best in vitro antifungal activity, while fluconazole was ineffective against *S*. *schenckii*.

### 3.3. Molecular Identification

Molecular identification was done by CAL gen PCR-sequencing. Thirty-eight were identified as *Sporothrix schenckii* and one as *Sporothrix globosa*, which came from the city of General Zaragoza, Nuevo León and showed a lymphocutaneous clinical presentation. Their geographical origin was as follows: Mexico City (43.5%), Oaxaca (15.3%), Puebla (10.2%), Nuevo León (7.6%), San Luis Potosí (7.6%), Veracruz (5.1%), Coahuila (5.1%), and Jalisco (5.1%). The most prevalent clinical presentation was lymphocutaneous sporotrichosis (92.3%), followed by fixed cutaneous sporotrichosis (5.1%), and one case of disseminated sporotrichosis (2.5%) (Table 1). Genetic identification was done using a fragment of the CAL gene, ~785 base pairs (bp). Our results showed diverse geographic distribution of clinical isolates in eight states. Thirty-nine isolates were divided into five supported clades. Clade I consisted of 20 isolates: Mexico City (9), Puebla (3), Oaxaca (3), Jalisco (2), Veracruz (2), and Nuevo León (1). Clade II consisted of 10 isolates: Mexico City (6), Coahuila (2), Puebla (1), and San Luis Potosi (1). Clade III consisted of five isolates: Oaxaca (2), Puebla (1), San Luis Potosi (1), Nuevo Leon (1). Clade IV consisted of three isolates: Mexico City (1), Oaxaca (1), and San Luis Potosi (1). Clade V consisted of one isolate from Nuevo Leon.

**Figure 3:**
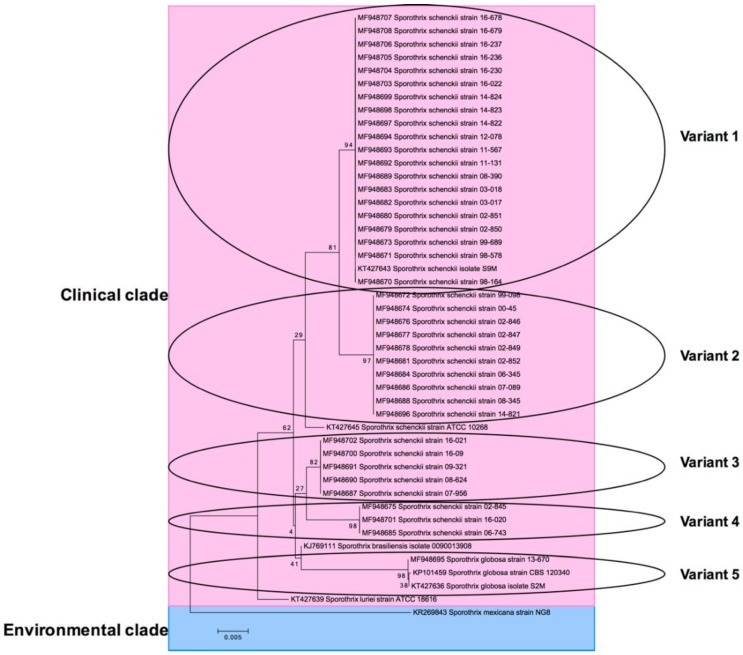
Phylogenetic tree of the CAL coding sequences from various fungi. The tree was built using MEGA version 6.06 by the neighbor-joining (NJ) method and further bootstrap analysis of 1000 replicas. Number on the branches indicate the bootstrap value. From the top to the bottom, clades are in linage-specific manner. Pink encompasses the clinical species, while blue is the environmental specie that was used as the out-group. Clinical clade was further divided into small subclades or variants (ovals) that correspond to the specific *Sporothrix* specie. Subclades did not match with geographical origin or antifungal resistance.

BLAST analysis showed the following identity: Clade (I) KT427643 = MF948670, MF948671, MF948673, MF948679, MF948680, MF948682, MF948683, MF948689, MF948692, MF948693, MF948694, MF948697, MF948698, MF948699, MF948703, MF948704, MF948705, MF948706, MF948707, and MF948708; Clade (II) MF948696 = MF948672, MF948674, MF948676, MF948677, MF948678, MF948681, MF948684, MF948686, and MF948688; Clade (III) MF948700 = MF948687, MF948690, MF948691, and MF948702; Clade (IV) MF948701 = MF948675 and MF948685; and Clade (V) MF948695 = KT427636 and KP101459. Thus, there were only five genetic clades according to the CAL gene—I, II, III, IV, and V. The clade arrangement in a lineage-specific manner is shown in Figure 3. From top to bottom, *S*. *schenckii*, *S*. *brasiliensis*, *S*. *globosa*, and *S*. *luriei* branches can be seen. Finally, *S*. *mexicana* was used as the out-group. Also to note, *S*. *schenckii* was subdivided into two subclades: (1) KT427643, MF948670, and MF948696; and (2) MF948700 and MF948701.

## 4. Discussion

The species of *Sporothrix* was previously described as a single species. However, it is now recognized as *S*. *schenckii* complex based on phylogenetic studies using DNA sequence in specific regions of rDNA, internal transcribed spacer (ITS), calmodulin, β-tubulin, and translation elongation factor 1 genes. Calmodulin gene in particular has been widely used for taxonomy for *Sporothrix* species [5,10]. In our study, 39 isolates were identified by partial sequences of the calmodulin gene. The phylogenetic tree showed five main branches (variants). These variants matched with *Sporothrix schenckii* CAL gene sequences as expected, but their phylogenetic relationship was not related to their geographical origin. For example, variant 4 had clinical isolates from Mexico City (strain 02-845), Oaxaca (strain 16-020), and San Luis Potosi (strain 06-743). One isolate (MF948695) was clustered into the *S*. *globosa* clade. In a recent study, *S*. *schenckii sensu stricto* was divided in five subclades with calmodulin for AFLP and phylogenetic analysis of isolates from all over the world [6]. Suzuki et al., concluded that *Sporothrix* species have a distinct virulence profile, but there is no clear relationship between genotypes *S*. *globosa* subgroups I, II, or *S*. *Schenckii sensu stricto* and geography in Japan or clinical forms [5]. The disease is characterized by nodular cutaneous and subcutaneous lesions, which may involve the adjacent lymphangitic system. The most common clinical manifestation was lymphocutaneous in this study, which is in agreement with the literature [4,7,11,12,20]. The results showed that only one isolate was identified as *S*. *globosa*. This species was reported in 2006 by Marimon et al. [12]. *S*. *globosa* is the most extensively studied species, with a report on North, Central and South America, Europe, and Asia [21]. Other reports in our country have revealed two species that are etiological agents of human sporotrichosis [2,7]. However, *S*. *schenckii sensu stricto* is the most common species that causes sporotrichosis in Mexico [2]. The geographic origin of *Sporothrix* species in Mexico is diverse [7]. A recent study of 22 Mexican isolates found San Luis Potosi as the principal origin. This was contrary to Chakrabarti et al. who reported Puebla as a high endemic region [2]. We found that most isolates were from Mexico City, followed by Oaxaca. This does not correspond with the recent report from Rangel-Gamboa et al., which found clinical isolates from San Luis Potosi, followed by Puebla. However, the most frequent clinical manifestation was lymphocutaneous, which conformed to our finding. The distribution of *S*. *globosa* has been reported in many countries [4,5,6,8,13,21,22]. A few reports of clinical presentations of this species exist; most presentations were lymphocutaneous, with fixed cutaneous being a less frequent disseminated presentation [4,8,22]. In this work, lymphocutaneous was the most frequent presentation. Our clinical isolate identified as *S*. *globosa* (MF948695) had a lymphocutaneous sporotrichosis. This isolate grew well at both 30 °C and 35 °C and the growth was restricted at 37 °C; this was the same results reported by Camacho et al. [22]. Treatment for sporotrichosis depends on the clinical manifestation; intolerance to iodine and the high toxicity of amphotericin B limits its use. In the last decades, azole derivatives have been the most effective drugs [23]. Antifungal susceptibility testing varies substantially in reports of in vitro susceptibility from human clinical isolates. We documented susceptibility of the in vitro profile of *Sporothrix schenckii* complex with molecular identification from China, Japan, Iran, Brazil, and Argentina [5,9,21,22,24,25]. In the present study, we evaluated nine antifungal agents against the mycelial phase of *S*. *schenckii* complex and showed that terbinafine was the most effective drug with a low MIC, while fluconazole was less active; this was comparable to other studies [9,25,26,27]. Recently Cordoba et al. described similar results for *S*. *schenckii* and *S*. *globosa* in mycelial phase in Argentina [25]. The behavior of *S*. *globosa* was equal to other isolates in this study.

## 5. Conclusions

The major species involved in human sporotrichosis are *S*. *schenckii*, *S*. *brasiliensis*, and *S*. *globosa* of diverse geographic origin. Sporotrichosis is common in regions of Latin America with tropical climates. In Mexico, it is a relevant mycosis, mainly in farmers who work with a variety of vegetation [7]. *S*. *schenckii* has revealed a high genetic diversity and a recent population expansion process, while *S*. *globosa* and *S*. *brasiliensis* has shown a rapid clonal distribution [8]. The isolates studied in this paper revealed a high degree of genotypic variability among *S*. *schenckii sensu stricto* isolates. Based on geographic origin, *S*. *schenckii* complex have a wide geographical distribution in our country. Our study showed there was no significant difference in the antifungal in vitro susceptibility among *Sporothrix* species, including *S*. *globosa* in filamentous phase. This study of *S*. *globosa* represents the first report of a clinical isolate from Northern Mexico.

## Figures and Tables

**Table 1:** Data of clinical human sporotrichosis and molecular analysis used in this study.

Strain	Species (Molecular Identification)	Lesion Type	Origin from Mexico	GenBank Accession No. CAL Gen	Variant in Phylogenetic Tree
98-164	*Sporothrix schenckii*	Lymphocutaneous	Mexico City	MF948670	1
98-578	*Sporothrix schenckii*	Lymphocutaneous	Mexico City	MF948671	1
99-098	*Sporothrix schenckii*	Lymphocutaneous	Mexico City	MF948672	2
99-689	*Sporothrix schenckii*	Fixed cutaneous	Mexico City	MF948673	1
00-45	*Sporothrix schenckii*	Lymphocutaneous	Mexico City	MF948674	2
02-845	*Sporothrix schenckii*	Lymphocutaneous	Mexico City	MF948675	4
02-846	*Sporothrix schenckii*	Lymphocutaneous	Mexico City	MF948676	2
02-847	*Sporothrix schenckii*	Lymphocutaneous	Mexico City	MF948677	2
02-849	*Sporothrix schenckii*	Fixed cutaneous	Mexico City	MF948678	2
02-850	*Sporothrix schenckii*	Lymphocutaneous	Mexico City	MF948679	1
02-851	*Sporothrix schenckii*	Lymphocutaneous	Mexico City	MF948680	1
02-852	*Sporothrix schenckii*	Lymphocutaneous	Mexico City	MF948681	2
03-017	*Sporothrix schenckii*	Lymphocutaneous	Veracruz	MF948682	1
03-018	*Sporothrix schenckii*	Lymphocutaneous	Veracruz	MF948683	1
06-345	*Sporothrix schenckii*	Lymphocutaneous	San Luis Potosi	MF948684	2
06-743	*Sporothrix schenckii*	Lymphocutaneous	San Luis Potosi	MF948685	4
07-089	*Sporothrix schenckii*	Lymphocutaneous	Coahuila	MF948686	2
07-956	*Sporothrix schenckii*	Lymphocutaneous	San Luis Potosi	MF948687	3
08-345	*Sporothrix schenckii*	Lymphocutaneous	Coahuila	MF948688	2
08-390	*Sporothrix schenckii*	Lymphocutaneous	Mexico City	MF948689	1
08-624	*Sporothrix schenckii*	Lymphocutaneous	Nuevo Leon	MF948690	3
09-321	*Sporothrix schenckii*	Lymphocutaneous	Mexico City	MF948691	3
11-131	*Sporothrix schenckii*	Lymphocutaneous	Puebla	MF948692	1
11-567	*Sporothrix schenckii*	Lymphocutaneous	Nuevo Leon	MF948693	1
12-078	*Sporothrix schenckii*	Lymphocutaneous	Oaxaca	MF948694	1
13-670	*Sporothrix globosa*	Lymphocutaneous	Nuevo Leon	MF948695	5
14-821	*Sporothrix schenckii*	Lymphocutaneous	Puebla	MF948696	2
14-822	*Sporothrix schenckii*	Lymphocutaneous	Puebla	MF948697	1
14-823	*Sporothrix schenckii*	Lymphocutaneous	Puebla	MF948698	1
14-824	*Sporothrix schenckii*	Lymphocutaneous	Oaxaca	MF948699	1
16-09	*Sporothrix schenckii*	Lymphocutaneous	Oaxaca	MF948700	3
16-020	*Sporothrix schenckii*	Lymphocutaneous	Oaxaca	MF948701	4
16-021	*Sporothrix schenckii*	Disseminated	Oaxaca	MF948702	3
16-022	*Sporothrix schenckii*	Lymphocutaneous	Oaxaca	MF948703	1
16-230	*Sporothrix schenckii*	Lymphocutaneous	Mexico City	MF948704	1
16-236	*Sporothrix schenckii*	Lymphocutaneous	Mexico City	MF948705	1
16-237	*Sporothrix schenckii*	Lymphocutaneous	Mexico City	MF948706	1
16-678	*Sporothrix schenckii*	Lymphocutaneous	Jalisco	MF948707	1
16-679	*Sporothrix schenckii*	Lymphocutaneous	Jalisco	MF948708	1

**Table 2:** The results of MIC_50_ and MIC_90_ of nine antifungal drugs against 39 isolates of *S*. *schenckii* complex.

Antifungal	GM	Range (µg/mL)	MIC_50_ (µg/mL)	MIC_90_ (µg/mL)
Amphotericin B AMB	2.554	0.5–4	4	4
Itraconazole ITC	4	0.125–2	4	8
Voriconazole VRC	2.946	0.5–8	2	4
Posaconazole PSC	1.769	0.5–8	1	8
Fluconazole FLC	>64	64–>64	64	>64
Terbinafine TRB	0.374	0.06–1	0.5	1
Caspofungin CSF	1.959	0.5–8	2	4
Anidulafungin ANF	2.083	0.25–8	2	4
Micafungin MCF	2.402	0.5–8	2	4

MIC_50_-MIC_90_: minimal inhibitory concentration at which 50% and 90% of microorganisms are inhibited. GM: geometric mean.
